# Genetic Diversity and Connectivity of Reef-Building *Halimeda macroloba* in the Indo-Pacific Region

**DOI:** 10.3390/plants14101497

**Published:** 2025-05-16

**Authors:** Xiaohan Song, Jianting Yao, Michael Y. Roleda, Yanshuo Liang, Rui Xu, Yude Lin, Shienna Mae C. Gonzaga, Yuqun Du, Delin Duan

**Affiliations:** 1 State Key Laboratory of Breeding Biotechnology and Sustainable Aquaculture, Institute of Oceanology, Chinese Academy of Sciences, Qingdao 266000, China; 2 Laboratory for Marine Biology and Biotechnology, Qingdao Marine Science and Technology Center, Qingdao 266200, China; 3 Algal Ecophysiology (AlgaE) Laboratory, Marine Science Institute, University of the Philippines, Quezon City 1101, Philippines; 4 Laboratory of Marine Organism Taxonomy and Phylogeny, Qingdao Key Laboratory of Marine Biodiversity and Conservation, Institute of Oceanology, Chinese Academy of Sciences, Qingdao 266071, China; 5 Melbourne Integrative Genomics, School of Biosciences, University of Melbourne, Melbourne, VIC 3010, Australia

**Keywords:** population structure, gene flow, ocean currents, calcified algae, seaweed conservation

## Abstract

Understanding population genetic connectivity is crucial for the sustainability and persistence of marine biodiversity. As a fundamental reef-building macroalga of the coastal ecosystem, *Halimeda macroloba* Decaisne is one of the dominant intertidal seaweeds in the Indo-Pacific region. However, its genetic structure and population connectivity have been poorly recognized. Here, we explored the population genetic structure and genetic connectivity of *H. macroloba* using chloroplast *tuf*A, *rps*3-*rpl*14, and *rbc*L. Our results indicated low genetic diversity and shallow population genetic structure at the intraspecific level, uncovering five genetic groups with six subdivided lineages in *tuf*A and two genetic clusters in *rps*3-*rpl*14. We detected demographic expansion in the last glacial period of the Pleistocene and significantly asymmetric gene flow among different geographical units. We suggest that the southwestward ocean currents under the influence of northeast monsoon in the Indo-Pacific region are the main factor in shaping the present genetic structure, and the asexual reproduction of *H. macroloba* also plays an important role of the low genetic diversity pattern; in addition, the divergence between genetic clusters might be related to the historical isolation led by the paleoclimate oscillation in the Pleistocene. The Xisha Islands in the northern South China Sea might serve as a potential refugium of *H. macroloba*, which needs extra attention to conservation management. Given the limitation of sample size, we need to conduct more field work and carry out further research at a larger scale in the future. Our study provided new insights into the theory of population connectivity in the Indo-Pacific region and provided scientific basis for tropical costal seaweed conservation.

## 1. Introduction

Connectivity conservation is essential for maintaining biodiversity and adapting to climate change across all biomes and spatial scales [1,2]. Increasing connectivity can increase ecosystem resilience and facilitate population recovery [3]. Compared with terrestrial ecosystems, marine ecosystems have higher dynamic and lower spatial heterogeneity, and it is difficult to distinguish habitat patches between different ecosystems, which undoubtedly increases the difficulty of biological connectivity research and application [4]. In recent years, intensive anthropogenic activities and climate change have led to the loss and fragmentation of coastal ecosystems [5], which has forced us to accelerate research on coastal connectivity to develop better conservation strategies.

As a crucial producer of the coastal ecosystem, seaweeds possess substantial economic, ecological, and social value, providing extensive resources such as food, medicine, oxygen, shelter, and habitat for both humans and marine organisms [6]. As highly calcareous green algae, the genus *Halimeda* (Bryopsidales, Chlorophyta) serves as one of the most important reef-building macroalgae in the global coastal coral reef ecosystem [7,8,9]. Recent studies have revealed that the *Halimeda* species have an intricate evolutionary history related to the paleoclimate oscillation in the Indo-Pacific region [10,11], which is a well-known hotspot of marine biodiversity around the world with complex geological history, coastal topography, hydrological conditions, and marine environment [12]. However, the research on intraspecific genetic connectivity of *Halimeda* species in tropical coastal waters is still lacking. Consequently, studying the genetic connectivity of *Halimeda* species can provide insight into the conservation of calcified algae resources in the Indo-Pacific region and provide scientific evidence for the protection of tropical coral reef ecosystems.

Among *Halimeda* species, *Halimeda macroloba* Decaisne acts as an important carbonate contributor in the tropical intertidal ecosystem [13]. *Halimeda macroloba* is widely distributed in the Indo-Pacific region and functions as the dominant species around the Thai–Malay Peninsula, with the distribution pattern affected by the water depth, sea surface temperature, and phosphate concentration [14]. Previous studies have demonstrated that it seemed to harbour low genetic variation in the Indo-Pacific region [15,16], but the genetic connectivity at the intraspecific level remains poorly acknowledged.

The chloroplast genetic markers were extensively employed for phylogenetic and phylogeographic studies of the macroalgae belonging to Bryopsidales due to the advantages of high sequence conservation and stability of maternal inheritance [17,18,19]. In order to acquire broader molecular data and easier integration of published data, we selected three chloroplast markers (*tuf*A, *rps*3-*rpl*14, and *rbc*L) for amplification experiments, which have been utilized in many studies of *Halimeda* species [20,21,22]. In this study, we investigated the genetic diversity and population structure of *H. macroloba* in the Indo-Pacific region and estimated the genetic connectivity mechanism maintaining present distribution patterns at the intraspecific level. Our results could enrich the tropical seaweed genetic theory and further our understanding of the biodiversity conservation of reef-building macroalgae.

## 2. Results

### 2.1. Genetic Diversity and Population Genetic Structure

**Figure 1:**
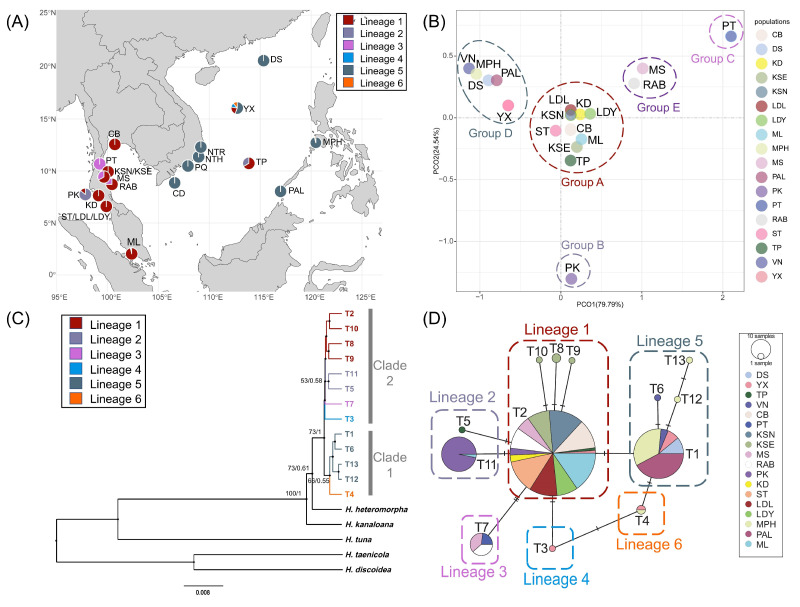
The phylogeographical structure of *H. macroloba* in *tuf*A. (**A**) The geographical distribution of 6 lineages in the Indo-Pacific region. (**B**) Population genetic structure based on PCoA analysis. (**C**) Phylogenetic relationship of *H. macroloba* based on *tuf*A haplotypes. Numbers above or near branches are Maximum likelihood bootstrap values (**left**)/Bayesian posterior probabilities (**right**). Values < 50% or <0.50 are not shown. (**D**) Median-joining network based on *tuf*A haplotypes. The circle size represents the sample size. The number of bars on the connecting lines represents the genetic steps.

We obtained 308 *tuf*A sequences and 181 *rps*3-*rpl*14 sequences, with 13 and 6 haplotypes, respectively (Table S1). Unexpectedly, the amplification of *rbc*L was challenging, and we eventually obtained 43 sequences and two haplotypes (Table S1). Apart from two Chinese populations (YX and TP) which displayed moderate haplotype diversity in *tuf*A, all other populations exhibited the pattern of low haplotype diversity and low nucleotide diversity (Table S1). Because of the limited sample size of four Vietnamese sites (Table S1), we combined the Vietnamese sequences as one population named VN (Figure 1).

Due to the limited number of *rbc*L sequences available for population analysis, we utilized the *tuf*A and *rps*3-*rpl*14 datasets to investigate the population genetic structure. In *tuf*A, congruent results were found both in the phylogenetic relationship and haplotype network, which indicated two main clades and six sub-lineages at the intraspecific level (Figure 1C,D). Specifically, lineage 1 was mainly distributed along the Thai–Malay Peninsula; lineage 2 only occurred in Phuket in Thailand and Taiping Island in China; lineage 3 was restricted to along the Gulf of Thailand; lineage 5 was widely found along the coast of the South China Sea; the two small remaining lineages (lineage 4 and lineage 6) were limited to the Xisha Islands of China (Figure 1A). The YX population consisted of haplotypes from four lineages, acting as the place with richest genetic diversity in the studying area (Figure 1A; Table S1). The population genetic structure by PCoA analysis was slightly different from the haplotype network, which demonstrated five genetic groups. The populations along the coast of the Thai–Malay Peninsula and the South China Sea belonged to distinct genetic groups (group A and group D) (Figure 1B). Additionally, the Phuket population (PK) and Pi-Tak population (PT) in Thailand formed independent genetic groups, respectively, whereas the two Surat Thani populations (MS and RAB) along the Gulf of Thailand constituted a single genetic group collectively (Figure 1B).

**Figure 2:**
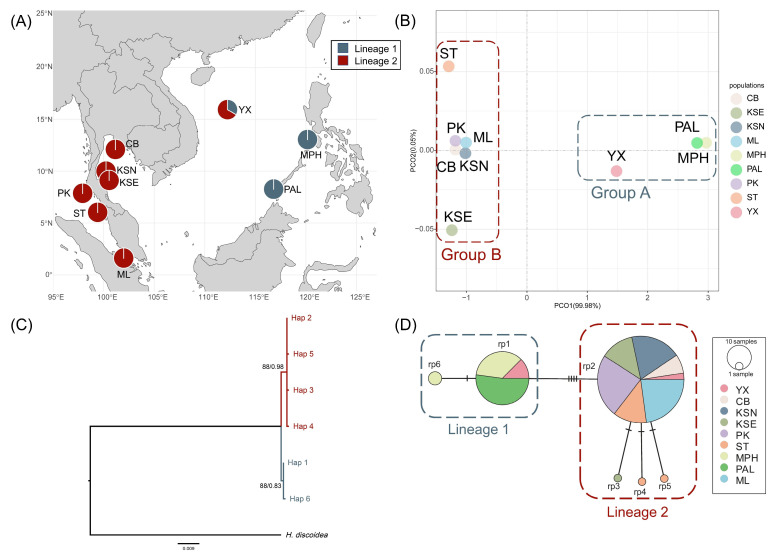
The phylogeographical structure of *H. macroloba* in *rps*3-*rpl*14. (**A**) The geographical distribution of 2 lineages in the Indo-Pacific region. (**B**) Population genetic structure based on PCoA analysis. (**C**) Phylogenetic relationship of *H. macroloba* based on *rps*3-*rpl*14 haplotypes. Numbers above or near branches are Maximum likelihood bootstrap values (**left**)/Bayesian posterior probabilities (**right**). Values < 50% or <0.50 are not shown. (**D**) Median-joining network based on *rps*3-*rpl*14 haplotypes. The circle size represents the sample size. The number of bars on the connecting lines represents the genetic steps.

In *rps*3-*rpl*14, concordant patterns were observed across the population genetic structure, phylogenetic relationship, and the haplotype network, which indicated two genetic clusters at the intraspecific level (Figure 2). Group A occurred in Chinese and Philippine populations (YX, MPH, and PAL), and group B covered the entire Thai–Malay Peninsula and the northern South China Sea (Figure 2).

**Figure 3:**
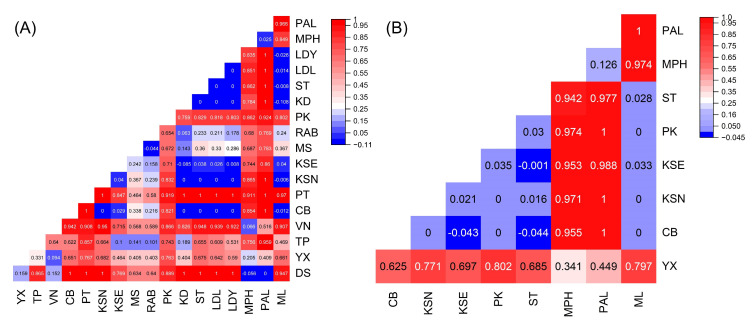
Pairwise genetic differentiation (*F*_st_) between populations based on *tuf*A (**A**) and *rps*3-*rpl*14 (**B**). Values less than 0.25 were indicated in blue, and values greater than 0.25 were indicated in red.

In order to examine the genetic differentiation of *H. macroloba*, we conducted analysis of molecular variance (AMOVA) and analysis of the pairwise fixation index (*F*_st_) both on *tuf*A and *rps*3-*rpl*14. The AMOVA results showed that the genetic differentiation mainly occurred among genetic groups and within populations (Table S2). The pairwise *F*_st_ of *tuf*A suggested that most populations had obvious genetic differentiation, and in particular, the differentiations between populations belonging to different genetic groups were more significant (Figure 3A). The pairwise *F*_st_ of *rps*3-*rpl*14 showed consistency with the genetic structure, indicating that significant genetic differentiation was observed between populations in the South China Sea (group A) and populations along the Thai–Malay Peninsula (group B), whereas no considerable differentiation was detected among populations within the same group (Figure 3B).

### 2.2. Demographical Population History

**Figure 4:**
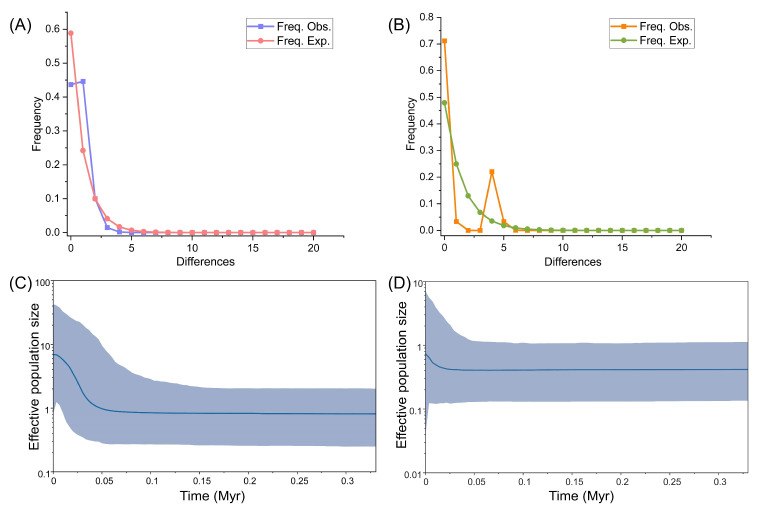
Demographic dynamics of *H. macroloba* in *tuf*A and *rps*3-*rpl*14. (**A**) Mismatch distribution of *tuf*A. (**B**) Mismatch distribution of *rps*3-*rpl*14. (**C**) BSP results of *tuf*A. The horizontal axis represents the time in the past in the unit of million years (Myr) ago, and the vertical axis represents the effective population size. The blue intervals represent the 95% highest posterior density (HPD). The blue curve represents the median value. (**D**) BSP results of *rps*3-*rpl*14. The horizontal axis represents the time in the past in the unit of million years (Myr) ago, and the vertical axis represents the effective population size. The blue intervals represent the 95% highest posterior density (HPD). The blue curve represents the median value.

With the timeframe proposed by Verbruggen et al. (2009) [23], the *Halimeda* genus diverged at about 144 Myr before present. Therefore, we calibrated that the molecular clock of *tuf*A and *rps*3-*rpl*14 as 0.093–0.095%/Myr and 0.108–0.11%/Myr, respectively. Mismatch analysis showed unimodal distribution patterns based on *tuf*A and *rps*3-*rpl*14, which supported the recent demographical expansion model (Figure 4A,B). Furthermore, in Bayesian skyline plot (BSP) analyses, the intraspecific dynamics initiated approximately 0.363 Myr ago both in *tuf*A and *rps*3-*rpl*14 (Figure 4C,D). Corresponding to the results of mismatch analysis, demographic expansions were detected in both markers. In *tuf*A, we found significant expansion from 0.05 Myr ago (Figure 4C); in *rps*3-*rpl*14, we found that *H. macroloba* remained stable during a long period and then expanded slightly from about 0.025 Myr ago (Figure 4D). In addition, we conducted a neutral test (Tajima’s *D* and Fu’s *F*_s_) of *tuf*A and *rps*3-*rpl*14 at the population level. Although the *p* value of most results was not significant, we found potential expansion in some populations (KSE, ST, MPH, and ML) and potential contraction in several others (YX, MS, RAB, and PK) (Table S3).

### 2.3. Population Gene Flow

**Figure 5:**
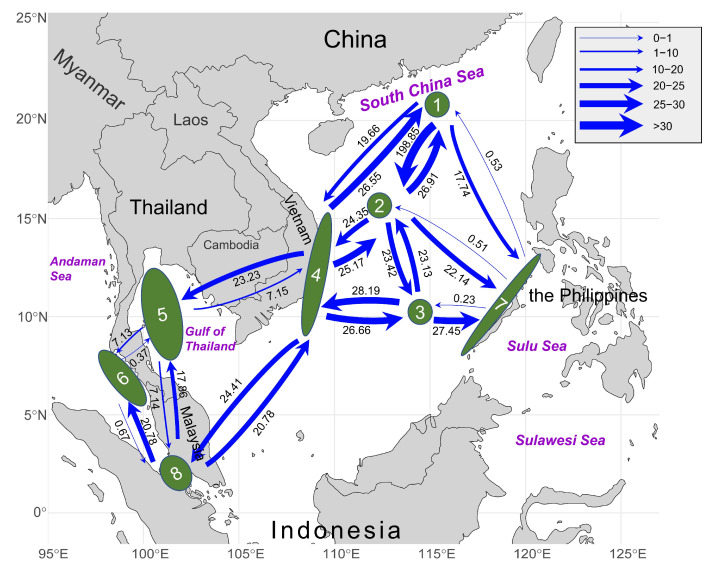
Gene flow (*Nm*) between 8 sub-units based on *tuf*A. Numbers above/below the arrows represent migration rates in the direction of the arrow. Arrow thickness was scaled according to the value.

We used the *tuf*A dataset to infer the population gene flow (*Nm*) due to more extensive geographical populations than *rps*3-*rpl*14 dataset. The Bayesian migration analysis inferred from *tuf*A revealed asymmetric gene flow among eight sub-units in the Indo-Pacific region (Figure 5). On the one hand, we found a high level of genetic exchange in the vast South China Sea region, including three Chinese populations (1, 2, and 3), Vietnamese populations (4), and Philippine populations (7). Strong bidirectional gene flow was detected among Chinese and Vietnamese units, and the greatest gene flow was found from the Dongsha Islands to the Xisha Islands (1 → 2: *Nm* = 198.85). However, all the gene flows from the Philippine unit to other units were extremely low. On the other hand, asymmetric gene flow occurred between the populations in the South China Sea region and the populations in the Gulf of Thailand (5), the Andaman Sea (6), and the Malacca Strait (8). Southwestward gene flow from Vietnam (4) to the Gulf of Thailand (5) was significantly higher than that in the opposite direction (4 → 5: *Nm* = 23.23; 5 → 4: *Nm* = 7.15) (Figure 5). Meanwhile, gene flow from Vietnam (4) to the Malacca Strait (8) was also fractionally higher than that in the opposite direction (4 → 8: *Nm* = 24.41; 8 → 4: *Nm* = 20.78) (Figure 5). Similarly, the southwestward genetic exchange from the Gulf of Thailand to the Andaman Sea was obviously higher than that in the opposite direction (5 → 6: *Nm* = 7.13; 6 → 5: *Nm* = 0.37). Notably, we found high genetic exchange from the Malacca Strait (8) to the Andaman Sea (6) and the Gulf of Thailand (5) (Figure 5).

## 3. Discussion

### 3.1. Genetic Connectivity Derived by Ocean Currents

Genetic diversity serves as a fundamental pillar for ecosystem resilience, facilitating species adaptation to environmental changes, and underpinning nature’s essential contributions to humanity [24,25]. Among marine organisms, population connectivity has a significant impact on the differentiation and population genetic structure [26]. Ocean currents can facilitate gene flow between populations to promote genetic homogeneity; conversely, they can also lead to increased genetic differentiation among populations as physical barriers [27,28,29]. Based on the three chloroplast molecular datasets, a pattern of low genetic diversity and high homogeneity within *H. macroloba* was displayed generally (Figure 5, Table S1). Meanwhile, we discovered a shallow population genetic structure and strong gene flow at the intraspecific level (Figure 1, Figure 2, and Figure 5), which was contrary to the general notion that *Halimeda* species had limited dispersal [10,22]. The species exhibiting high fecundity and dispersal capabilities usually lack significant spatial genetic structure [30]. Although mature *Halimeda* thalli are unlikely to be long-distance travellers due to the characteristic of strong calcification and rapid sinking as sediment [31], previous research has proposed that the propagules and the juvenile uncalcified thalli might act as the main force of dispersal [32]. Therefore, the phylogeographical pattern of *H. macroloba* was likely caused by the influence of ocean currents in the Indo-Pacific region.

Our integrated results suggested that the gene flow directionality corresponded to the southwestward ocean currents in the Indo-Pacific region. In the northern and central parts of the South China Sea, the bidirectional gene flow was strong between populations (1, 2, 3, and 4), and there was little gene flow from the eastern coast of the South China Sea to the northern or central South China Sea (Figure 5). Meanwhile, the gene flow from the populations of the South China Sea to populations of the Gulf of Thailand and the Andaman Sea exhibited a significant southwestward pattern (Figure 5). Such a genetic connectivity pattern might be mediated by the southwest surface currents in this region. Affected by the seasonal monsoon climate in the Indo-Pacific region, the ocean current system was complicated and intense, characterized by opposite flow directions in winter and summer; the surface circulation in winter flows mainly from northeast to southwest, which could facilitate the genetic exchange at the intraspecific level [33]. During the winter monsoon period, the South China Sea throughflow (SCSTF) enters the South China Sea through the Luzon Strait and flows westward and southward along the northern boundary of the South China Sea, while the current on the eastern side is weaker; meanwhile, a branch from the northwestern direction flows southeastward into the Mindoro Strait [34]. This might explain the strong gene flows toward the southwest from the populations of the South China Sea to the populations of the Thai–Malay Peninsula and reveal the reason why the gene flow from the eastern edge of the South China Sea to the central and northern regions was weaker. Additionally, the strong bidirectional gene flow in the northern and western South China Sea might result from the comprehensive influence of ocean surface circulations changing direction throughout the year and stepping stones provided by numerous islands [35,36].

Remarkably, we found obvious gene flow toward the north along the coast of the Malacca Strait (Figure 5). This might be related to the hydrological conditions of the Malacca Strait during the winter monsoon as well. Located within the equatorial doldrums, the current of the Malacca Strait is influenced by the equatorial low-pressure zone and the topography of the coastline, and due to the impact of the winter monsoon, the winter surface current in the Malacca Strait generally moves from the southeast to the northwest towards the Andaman Sea (flowing from areas of higher to lower water volume) [37], which corresponds to the stronger northward gene flow from units 8 to 6 (Figure 5).

Similar conditions were documented in other seaweed species and adjacent seas. For *Sargassum* species, which are widespread with a high capability of dispersal (with air vesicles providing buoyancy), the ocean currents also played a dominant role in their genetic structure [38,39]; furthermore, the southwestern current in the Indo-Pacific region might act as a critical factor affecting their dispersal and colonization [40,41]. Our results demonstrated the importance of ocean currents in shaping the genetic connectivity of marine plants. Nevertheless, many studies have illustrated that the seaweeds in the Indo-Pacific region hold rich genetic diversity and a highly divergent structure, implying limited genetic exchange between different populations [42,43]; some research has indicated that genetic differentiation might arise from the topographical isolation and hydrological barrier of Thai–Malay Peninsula [44,45]. Therefore, extensive sample collection and further integrated studies are needed to shed light on the theory of seaweed genetic connectivity here.

### 3.2. Intraspecific Phylogeographical Pattern of H. macroloba

Based on the three chloroplast molecular datasets, the molecular diversity within *H. macroloba* displayed a pattern of low haplotype diversity and low nucleotide diversity (Table S1). However, we discovered more genetic variations in the *tuf*A dataset than *rps*3-*rpl*14, indicating that the *tuf*A gene marker might have a higher resolution in population genetic research and retain more historical relics in the evolutionary process. This result was in contrast to the findings of a previous study of Mediterranean *H. tuna* that more genetic variation was documented in *rps*2-*rpl*14 than *tuf*A [22]. This might be connected with the fact that the integrated data of *tuf*A in this study were more extensive than the *rps*3-*rpl*14 data. Meanwhile, we might obtain less genetic variation in the *rps*3-*rpl*14 dataset because of the shorter length of sequencing. Five genetic groups and six subdivided genetic lineages were found in *tuf*A (Figure 1B,C), and four lineages were located around the Xisha Islands (YX) in southern China (Figure 1A). Also, in *rps*3-*rpl*14, the YX population was composed of haplotypes containing both genetic lineages (Figure 2A). Moreover, the YX population displayed the highest haplotype diversity in this study (Table S1). The populations in glacial refugia usually retained higher genetic diversity and particular haplotypes than recolonized populations [46]. Hence, we hypothesized that the Xisha Islands might serve as a potential historical refugium for *H. macroloba*, which highlights the value of extra conservation and management here. But due to the limited sample size of this population, the reliability of the hypothesis still needs further study to be confirmed. In previous studies of other marine organisms, the Xisha Islands were regarded as a transfer station for genetic exchange rather than a diversity centre [47]. It is necessary to enlarge the collection range and quantity to ensure the accuracy of research results.

In addition, a previous study revealed that *Halimeda* species primarily reproduce asexually in their natural environment, and the abundant biomass on coral reefs is partially due to the ability of this genus to propagate asexually via vegetative fragmentation [31]. Thus, we assumed that the notably low genetic diversity of *H. macroloba* in the Indo-Pacific region may be associated with asexual reproduction (Table S1), and the separate genetic cluster in Phuket (PK) and Pi-Tak Island (PT) in Thailand might be the consequence of a local enrichment of one unique haplotype by vegetative fragmentation (Figure 1B).

Furthermore, the low genetic diversity and simple phylogeographical pattern could be correlated with historical vicariance caused by paleoclimate changes. Despite the lack of deep divergence, we still found genetic structures in different datasets (Figure 1 and Figure 2). Recent rapid expansion might lead to the simplification of genetic structure. The star-like haplotype network and unimodal distribution in mismatch together supported the population expansion, and the BSP directly showed the obvious expansion occurred during the Pleistocene (Figure 1D, Figure 2D and Figure 4). So, the present genetic structure may also be related to the historical demographical dynamics in the Pleistocene. Based on the BSP results, the divergence of this species in the Indo-Pacific region originated 0.363 Myr ago and then underwent expansion from 0.05 Myr to 0.025 Myr before present (Figure 4C,D), which occurred during the last glacial period in the Pleistocene. During this period, the extremely global cooling and ice sheet extension led to a significant drop in sea levels and large land exposure, resulting in the emergence of Sundaland and a reduction in the South China Sea, which became an inland sea only connected to the Pacific by the Bashi Strait during the Last Glacial Maximum [48,49,50]. Therefore, the division of dominant genetic lineages among the populations of the South China Sea and the Thai–Malay Peninsula (lineages 1 and 5 in *tuf*A, lineage 1 and 2 in *rps*3-*rpl*14) (Figure 1 and Figure 2) might arise from the long-term geographical isolation caused by the glacial climate oscillation, which resembled the cases that occurred in other *Halimeda* species. For example, Verbruggen et al. (2005) proposed that the distribution pattern of *H. cuneata* could be explained using the global cooling and isolation in the Plio-Pleistocene [51]; Rindi et al. (2020) represented a genetic break in *H. tuna* between the eastern and western Mediterranean Sea caused by the Pleistocene glaciation [22]; Zhang et al. (2024) discovered that the polyploidization timing of *H. opuntia* coincided with that of the sea level drop in the Pliocene [11]. Furthermore, we found respective unique lineages on east and west side of the Thai–Malay Peninsula (lineages 2 and 3 in *tuf*A) (Figure 1A), just like previous finding in other seaweeds [15,44,52]. The heterogeneity could also be attributed to the regional isolation by topography and historical isolation. This suggested that the ancestor of *H. macroloba* underwent a complex historical process during the last glacial period during the Pleistocene, and the current genetic structure may be the consequence of historical isolation and subsequent genetic mixture through sea level fluctuation [53,54,55]. These findings hinted at the necessity of considering the historical climate and geological events in research on seaweed genetic diversity distribution and evolutionary patterns.

### 3.3. Conservation and Management

The low genetic diversity and simple population genetic structure underscored the importance of genetic diversity protection of *H. macroloba*. We propose establishing two management units (MUs) in the South China Sea basin and the Thai–Malay Peninsula for separate conservation [56]. Moreover, given the higher haplotype diversity, probable population contraction risk, and potential glacial refugium of the Xisha population in China (Figure 1A, Tables S1 and S3), we suggest more attention should be paid to the management of seaweed source conservation in the Xisha Islands. Additionally, in view of variant genetic lineages on both sides of the Thai–Malay Peninsula, we recommend that separate management strategies should be considered for marine conservation.

## 4. Materials and Methods

### 4.1. Sample Collection, DNA Extraction, and Amplification

From 2019 to 2025, we collected *H. macroloba* samples in the Indo-Pacific convergence region along the coast of China, Thailand, Malaysia, and the Philippines (Table S1). Total genomic DNA was extracted using the Hi-DNAsecure Plant Kit (TIANGEN, Beijing, China, DP350-03) following the manufacturer’s instructions, and the quality was checked using 0.7% agarose gel electrophoresis and the OD260/OD280 ratio.

The chloroplast-encoded *tuf*A, *rps*3-*rpl*14, and *rbc*L were amplified as molecular markers. For *tuf*A, the amplification was carried out using the primer pairs *tuf*A-F (5′-TGAAACAGAAMAWCGTCATTATGC-3′) and *tuf*A-R (5′-CCTTCNCGAATMGCRAAWCGC-3′) [57] and the PCR procedures described in Cremen et al. (2016) [58]. For *rps*3-*rpl*14, the amplification was carried out with the primers *rps*3F (5′-ACACAACCGCATTTATCACA-3′) and *rpl*14R (5′-CAGCAACATTWACAYAACTTTCAG-3′) using the PCR procedures described in Rindi et al. (2020) [22]. For *rbc*L, the amplification was carried out with the primers U1-1 and U3-2 [59], and the PCR procedures were performed according to the procedures described by Curtis et al. (2008) [60]. The PCR products were checked in 1% agarose gel electrophoresis. Purification and sequencing of PCR products were performed using a BigDye Terminator Cycle sequencing kit and an ABI3730 automated sequencer (Applied Bio-systems, Foster City, CA, USA).

### 4.2. Molecular Diversity and Phylogenetic Relationship

To enlarge the sample size and geographical scale of the research, we integrated the newly discovered sequences in this study and published sequences available in GenBank and other studies (Table S4). The obtained sequence datasets were aligned with the MUSCLE model in MEGA X [61]. After manual adjusting for false or missing gaps, we computed the haplotype diversity, nucleotide diversity, variable sites, and parsimony informative sites for all populations in Arlequin 3.5.2.2 [62].

After testing the optimal substitution model of each dataset based on the Corrected Akaike Information Criterion (AICc) in jModelTest 2.1.7 [63], we reconstructed the phylogenetic relationships of each dataset based on maximum likelihood (ML) and Bayesian inference (BI). Phylogenetic ML trees were constructed based on 1,000 bootstrap replicates using a nearest-neighbour interchange heuristic method in PhyML 3.1 [64]. The BI trees were constructed based on 10,000,000 MCMC iteration chains, with the first 2,500,000 chains discarded as ‘burn-in’ in Beast 2.7.5 [65]. The convergence of the output tree dataset was checked in Tracer 1.7.1 [66] when the effective sample sizes (ESSs) > 200. The condensed tree was summarized in TreeAnnotator in Beast 2.7.5 with a 25% ‘burn-in’. The sequences of congeneric species *H. discoidea*, *H. heteromorpha*, *H. kanaloana*, *H. taenicola*, and *H. tuna* were selected as outgroups (Table S4). All trees were visualized using FigTree 1.4.4 (http://tree.bio.ed.ac.uk/software/Figtree/ accessed on 15 October 2024).

### 4.3. Population Genetic Structure and Differentiation

We estimated the population pairwise differentiation (*F*_st_) with 1000 permutations in Arlequin. The genealogic network diagrams based on the haplotypes of each dataset were drawn using the median-joining algorithm in POPART [67].

The population genetic structure of each dataset was detected using principal component analysis (PCoA) based on the population pairwise genetic distances in GenAlex 6.5.2.2 [68]. In addition, analysis of molecular variance (AMOVA) was conducted to explore the genetic differentiation of subdivisions based on the result of population genetic structure.

### 4.4. Divergence Time and Demographic History

We conducted the mismatch analysis using *tuf*A and *rps*3-*rpl*14 datasets in DnaSP v5.0 [69] to assess the historical population dynamics. We executed the neutral tests (Tajima’s *D* and Fu’s *F*_s_) of each population in Arlequin 3.5.2.2. We calibrated the molecular clock of *H. macroloba* using the timeframe proposed by Verbruggen et al. (2009) [23]. The divergence time of the *Halimeda* genus was estimated as 144 Myr ago, so we calculated the substitution rates of *tuf*A and *rps*3-*rpl*14 based on the genetic divergence between haplotypes of *Halimeda* species (*H. macroloba* and *H. discoidea*).

Aiming to study the population demographic history, the coalescent Bayesian skyline plot (BSP) analysis was performed in Beast v2.7.5 based on *tuf*A and *rps*3-*rpl*14. Molecular clocks of the two genes were chosen as relaxed clock log normal with calculated substitution rates. MCMC chains were set as 8 × 10^8^ iterations, followed by 2 × 10^8^ iterations discarded as burn-in, with sampling every 80,000 iterations.

### 4.5. Intraspecific Genetic Connectivity

In order to investigate the gene flow between populations, we estimated the migration rates and effective population size based on *tuf*A datasets in Migrate-n 4.4.3 [70]. We delineated 8 sub-units corresponding to the geographical distributions and population genetic structure (Figure 5). The analysis was conducted based on four long chains and ten replicates with a 10,000 ‘burn-in’ and a total of 50,000 steps along with sampling per 100 steps. Heating was set with four temperatures (1.0, 1.5, 2.5, and 5.0) with a static scheme.

## Data Availability

The newly discovered *tuf*A haplotypes of *H. macroloba* in this study are available in GenBank with the accession numbers PQ824573–PQ824582. The *rps*3-*rpl*14 haplotypes has been submitted to GenBank with a submission ID 2939450. The *rbc*L haplotypes have been submitted to GenBank with the submission ID 2939478. The newly discovered *tuf*A sequences of *H. discoidea* in this study are available in GenBank with the accession numbers PV368846–PV368848.

## Supplementary Materials

The following supporting information can be downloaded at: https://www.mdpi.com/article/10.3390/plants14101497/s1. Table S1: Molecular diversity inferred from chloroplast *tuf*A, *rps*3-*rpl*14, and rbcL of *Halimeda macroloba* in the Indo-Pacific region. Table S2: Analysis of molecular variance (AMOVA) based on *tuf*A and *rps*3-*rpl*14. Table S3: Neutral test of *Halimeda macroloba* populations based on *tuf*A and *rps*3-*rpl*14. Table S4: The *tuf*A sequences retrieved in GenBank and other research utilized in this study.

## Abbreviations

The following abbreviations are used in this manuscript:

AICc	Akaike Information Criterion
ML	Maximum likelihood
BI	Bayesian inference
ESS	Effective sample size
PCoA	Principal component analysis
AMOVA	Analysis of molecular variance
*F*_st_	Fixation index
BSP	Bayesian skyline plot
HPD	Highest posterior density
*Nm*	Migration rates
Myr	Million years
SCSTF	South China Sea throughflow
MU	Management unit
