# Supplementary material for: Genetic Diversity and Connectivity of Reef-Building Halimeda macroloba in the Indo-Pacific Region
Source: Plants (Basel). 2025 May 16;14(10):1497. doi: 10.3390/plants14101497 (PMC12115010; doi:10.3390/plants14101497)
Supplement: Supplementary file 1 [file plants-14-01497-s001.zip › plants-3571649-supplementary.pdf]

Table S1. Molecular diversity inferred from chloroplast *tuf*A, *rps*3-*rpl*14 and *rbc*L of *Halimeda macroloba* in the Indo-Pacific region

Country	ID	Sample sites	Coordinates	*tuf*A				*rps*3-*rpl*14				*rbc*L			
				n	N_h	*H*d	π (×10^{-2})	*n*	N_h	*H*d	π (×10^{-2})	*n*	N_h	*H*d	π (×10^{-2})
China	DS	Dongsha Islands, Sansha, Hainan	-	7	1	0	0	-	-	-	-	-	-	-	-
China	YX	Yongxing Island, Xisha Islands, Sansha, Hainan	16.83 °N, 112.33 °E	9	4	0.694±0.147	0.104±0.090	9	2	0.500±0.129	0.170±0.120	-	-	-	-
China	TP	Taiping Island, Gaoxiong, Taiwan	10.38 °N, 114.37 °E	3	2	0.667±0.314	0.078±0.097	-	-	-	-	-	-	-	-
Viet Nam	NTR	Nha Trang	12.22 °N, 109.2 °E	1	1	0	0	-		-	-	-	-	-	-
Viet Nam	NTH	Ninh Thuan	11.66 °N, 109.18 °E	1	1	0	0	-	-	-	-	-	-	-	-
Viet Nam	PQ	Phy Quy	10.55 °N, 108.96 °E	1	1	0	0	-	-	-	-	-	-	-	-
Viet Nam	CD	Con Dao, Ba Ria-Vung Tau	8.69 °N, 106.62 °E	1	1	0	0	-	-	-	-	-	-	-	-
Thailand	CB	SamaeSan village, Chon Buri	12.60 °N, 100.95 °E	21	1	0	0	9	1	0	0	-	-	-	-
Thailand	PT	Koh Pi-Tak, Chumphon	10.04 °N, 99.18 °E	3	1	0	0								
Thailand	KSN	Big Buddha, Koh Samui (North coast)	9.57 °N, 100.06 °E	25	1	0	0	24	1	0	0	1	1	0	0
Thailand	KSE	Koh Tean (East), Koh Samui, Suratthani	9.38 °N, 99.95 °E	20	4	0.363±0.131	0.046±0.050	17	2	0.118±0.101	0.010±0.019	1	1	0	0
Thailand	MS	Koh Mud Sum, Surat Thani	9.37 °N, 99.98 °E	15	2	0.476±0.092	0.111±0.090	-	-	-	-	-	-	-	-
Thailand	RAB	Koh Rab, Surat Thani	9.31 °N, 99.96 °E	20	2	0.395±0.101	0.092±0.078	-	-	-	-	-	-	-	-
Thailand	PK	Tangkhen Bay, Phuket	7.81 °N, 98.41 °E	35	1	0	0	30	1	0	0	11	1	0	0
Thailand	KD	Ko Kra Dan, Trang	7.31 °N, 99.26 °E	5	1	0	0	-	-	-	-	-	-	-	-
Thailand	ST	Koh LiDi, Satun	6.79 °N, 99.77 °E	24	1	0	0	18	3	0.216±0.124	0.019±0.026	4	1	0	0
Thailand	LDL	Koh Lidee Lek, Satun	6.78 °N, 99.77 °E	20	1	0	0	-	-	-	-	-	-	-	-
Thailand	LDY	Ko Lidee Yai, Satun	6.78 °N, 99.77 °E	15	1	0	0								
Malaysia	ML	Pulau Besar, Malacca	2.11 °N, 102.34 °E	30	2	0.067±0.061	0.008±0.018	29	1	0	0	8	2	0.250±0.180	0.084±0.072
the Philippines	MPH	Sablayan, Mindoro	12.86 °N, 120.75 °E	25	4	0.230±0.110	0.037±0.043	20	2	0.269±0.113	0.023±0.029	-	-	-	-
the Philippines	PAL	Balabac, Palawan, Philippines	8.26 °N, 117.17 °E	27	1	0	0	27	1	0	0	18	1	0	0

n, sample size; N_h, number of haplotypes; *H*d, haplotype diversity; π, nucleotide diversity; -, null.

Table S2. Analysis of molecular variance (AMOVA) based on *tuf*A and *rps*3-*rpl*14

	Among groups			Among populations within groups			Within populations		
	d.f.	%var	F_{ct}	d.f.	%var	F_{sc}	d.f.	%var	F_{st}
*tuf*A	4	78.74	0.79	13	0.81	0.04	290	20.45	0.80
*rps*3-*rpl*14	1	94.61	0.95	7	1.25	0.23	172	4.14	0.95

Table S3. Neutral test of *Halimeda macroloba* populations based on *tuf*A and *rps*3-*rpl*14

ID	Tajima's *D*		Fu's F_s	
	*tuf*A	*rps*3-*rpl*14	*tuf*A	*rps*3-*rpl*14
DS	0	-	0	-
YX	0.72	1.46	-1.28	3.78
TP	0	-	0.20	-
VN	-0.61	-	0.17	-
CB	0	0	0	0
PT	0	-	0	-
KSN	0	0	0	0
KSE	-1.44	-1.17	-2.14**	-0.75
MS	1.44	-	2.52	-
RAB	0.94	-	2.34	-
PK	0.06	0	0.53	0
KD	0	-	0	-
ST	0	-1.51*	0	-1.74*
LDL	0	-	0	-
LDY	0	-	0	-
MPH	-1.50*	-0.09	-2.44**	0.38
PAL	0	0	0	0
ML	-1.15	0	-1.21	0

*, $p < 0.05$; **, $p < 0.01$. -, null.

Table S4. The *tuf*A sequences retrieved in GenBank and other researches utilized in this study

Taxon	Accession numbers / Publications
Halimeda heteromorpha	AM049957
Halimeda kanaloana	AFP28146
Halimeda macroloba	MK922586
Halimeda macroloba	MN879375
Halimeda macroloba	MN879378
Halimeda macroloba	KU220836
Halimeda macroloba	KU220837
Halimeda macroloba	KU220838
Halimeda macroloba	KU220839
Halimeda macroloba	KU220840
Halimeda macroloba	KU220841
Halimeda macroloba	MK922585
Halimeda macroloba	OL422173
Halimeda macroloba	OL422175
Halimeda macroloba	OL422176
Halimeda macroloba	OL422177
Halimeda macroloba	Haplotype 1, Pongparadon et al. (2017)
Halimeda macroloba	Haplotype 2, Pongparadon et al. (2017)
Halimeda macroloba	Haplotype 3, Pongparadon et al. (2017)
Halimeda taenicola	OR861140
Halimeda tuna	MN935970
